# Transcriptional profiling of microglia; current state of the art and future perspectives

**DOI:** 10.1002/glia.23767

**Published:** 2019-12-17

**Authors:** Emma Gerrits, Yang Heng, Erik W. G. M. Boddeke, Bart J. L. Eggen

**Affiliations:** ^1^ Department of Biomedical Sciences of Cells & Systems, Section Molecular Neurobiology University of Groningen, University Medical Center Groningen Groningen The Netherlands

**Keywords:** human, microglia, mouse, single‐cell RNA‐sequencing, single‐nucleus RNA‐sequencing, transcriptomes

## Abstract

Microglia are the tissue macrophages of the central nervous system (CNS) and the first to respond to CNS dysfunction and disease. Gene expression profiling of microglia during development, under homeostatic conditions, and in the diseased CNS provided insight in microglia functions and changes thereof. Single‐cell sequencing studies further contributed to our understanding of microglia heterogeneity in relation to age, sex, and CNS disease. Recently, single nucleus gene expression profiling was performed on (frozen) CNS tissue. Transcriptomic profiling of CNS tissues by (single) nucleus RNA‐sequencing has the advantage that it can be applied to archived and well‐stratified frozen specimens. Here, we give an overview of the significant advances recently made in microglia transcriptional profiling. In addition, we present matched cellular and nuclear microglia RNA‐seq datasets we generated from mouse and human CNS tissue to compare cellular versus nuclear transcriptomes from fresh and frozen samples. We demonstrate that microglia can be similarly profiled with cell and nucleus profiling, and importantly also with nuclei isolated from frozen tissue. Nuclear microglia transcriptomes are a reliable proxy for cellular transcriptomes. Importantly, lipopolysaccharide‐induced changes in gene expression were conserved in the nuclear transcriptome. In addition, heterogeneity in microglia observed in fresh samples was similarly detected in frozen nuclei of the same donor. Together, these results show that microglia nuclear RNAs obtained from frozen CNS tissue are a reliable proxy for microglia gene expression and cellular heterogeneity and may prove an effective strategy to study of the role of microglia in neuropathology.

## Graphical abstract

- Microglia are heterogenous with regard to brain region, sex, age, and CNS disease.
- Microglia nuclear transcriptomes, from fresh and frozen CNS tissue, are a good proxy for freshly isolated microglia transcriptomes.

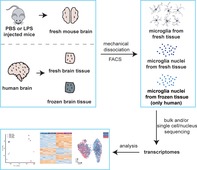

## 1 INTRODUCTION

Microglia are tissue macrophages in the central nervous system (CNS) that monitor homeostasis and are involved CNS disease (Butovsky & Weiner, 2018). As versatile macrophages with CNS‐specific functions, microglia can adopt a range of phenotypes, depending on the local neural microenvironment and stimulation type (De Biase et al., 2017; Gosselin et al., 2017). Over the last decade, gene expression profiling of purified microglia has greatly contributed to our understanding and characterization of these cells, both under normal and disease conditions. An overview of these studies will be provided in this review, which will conclude with a detailed description of recent technological developments on nuclear sequencing of microglia.

### 1.1 Mouse and human microglia transcriptomes by bulk population sequencing

The gene expression profile of mouse and human microglia was first identified using bulk population samples, for example, a large number of purified microglia in one sample. For mouse microglia, the first core microglia signature was generated in 2012 using microarrays in the Immunological Genome (ImmGen) project (Gautier et al., 2012). Based on this study, Chiu et al. compared microglia microarray data with data from 22 other myeloid cell types collected by the ImmGen project. Ninety‐nine genes were identified that were fivefold or more enriched in microglia relative to other myeloid immune cells (Chiu et al., 2013). Furthermore, they also compared spinal cord microglia RNA‐seq data with RNA‐seq data obtained from astroglia, motor neurons, and whole spinal cord, yielding 288 genes enriched in microglia. The overlap between two data sets identified 29 highly specific markers for microglia, including *Olfm3*, *Tmem119*, and *Siglech* (Chiu et al., 2013).

By direct RNA sequencing of sorted microglia and whole brain samples, Hickman et al. identified a cluster of genes responsible for mouse microglia sensing functions, referred to as the microglia sensome. Comparison with peritoneal macrophages identified 626 differentially expressed transcripts and the top 25 most highly expressed microglia transcripts include the sensome genes: *P2ry12*, *P2ry13*, *Tmem119*, *Gpr34*, *Siglech*, *Trem2*, and *Cx3cr1* (Hickman et al., 2013). These microglia signatures were confirmed in two studies that addressed the transcriptomic and epigenetic differences between mouse microglia and other tissue‐resident macrophages (Gosselin et al., 2014; Lavin et al., 2014). By gene profiling and quantitative mass spectrometry analysis, Butovsky et al. identified 1,572 genes and 455 proteins enriched in mouse microglia compared to CD11b^+^Ly6C^+^ spleen‐derived monocytes (Butovsky et al., 2014). Based on these two datasets, a Nanostring chip was designed to further investigate the differences between microglia and F4/80^+^ CD11b^+^ macrophages derived from peripheral organs. Two hundred thirty nine genes were specifically expressed by microglia and when compared to other CNS cells (astrocytes, oligodendrocytes, and neurons), 106 genes were microglia specific. *P2ry12*, *Fcrls*, *Tmem119*, *Olfml3*, *Hexb*, and *Tgfbr1* were identified as unique microglial genes; *PU.1* as a microglia‐specific transcription factor; and three microglia‐specific microRNAs (miR‐125b‐5p, miR‐342‐3p, and miR‐99a; Butovsky et al., 2014). Importantly, newborn microglia (P1), cultured primary microglia (P1‐2), microglia cell lines (N9, BV2), and embryonic stem cell‐derived microglia did not express these microglia signature genes (Butovsky et al., 2014).

Using microglia marker *Tmem119*, a mouse microglia gene expression profile during development and after an LPS challenge was generated (Bennett et al., 2016). During development, 37 of 100 top microglia‐enriched genes are consistently upregulated from E17 to P60. Again, a homeostatic microglia core signature was identified, and LPS induced a typical inflammatory gene profile.

All these studies led to the identification of a homeostatic microglia core gene expression signature, which includes S*all1*, *Hexb*, *Fcrls*, *Gpr43*, *Cx3cr1*, *Tmem119*, *Trem2*, *P2ry12*, *Mertk*, *Pros1*, and *Siglech*, genes that are abundantly expressed in microglia compared to other brain or myeloid cells.

In two studies published in 2017 (Galatro,Holtman, et al., 2017; Gosselin et al., 2017), the human microglia transcriptome was reported. Gosselin et al. expression profiled microglia isolated from surgically resected brain tissue of epilepsy, brain tumor, or acute ischemia patients. Microglia were isolated by Percoll gradient centrifugation and fluorescence‐activated cell sorting (FACS) on live‐CD11b^+^CD45^Low^CD64^+^CX3CR1^High^ cells, while excluding most activated cells with moderate to high levels of CD45. The 30 most abundant transcripts in microglia across different patients are related to known microglia properties and functions like ramification and motility (*P2RY12* and *CX3CR1*), synaptic remodeling (*C3* and *C1QA‐C*), and immune response (*HLA‐DRA* and *HLA‐B*) (Gosselin et al., 2017). Comparison of the microglia transcriptome to the transcriptome of cortical brain tissue used to isolate microglia, 881 microglia‐enriched (10‐fold increased) genes were detected (Gosselin et al., 2017).

In the study by Galatro et al., microglia were isolated from postmortem CNS tissues from donors without apparent neuropathological abnormalities. Microglia were isolated by mechanical dissociation, Percoll gradient centrifugation, and sorted as live‐CD11b^high^CD45^int^ cells. Compared to corresponding cortical tissue, 1,297 human microglial signature genes were detected (logFC >3 enrichment). Gene ontology (GO) analysis showed many significantly enriched terms and genes associated with the biological functions of microglia, such as immune signaling and modulation (*CD74*, *CSFR1*, *C1QA‐C*), pathogen and self‐recognition (*MyD88*, *CLECL1*, *CIITA*), and cell adhesion and motility (*ITGAM*, *CX3CR1*, *ICAM‐1*). Also, human microglia expressed many SIGLECs (*CD33*, *SIGLEC5/7*‐*12/14*), showing its important role for maintenance of CNS homeostasis (Galatro, Holtman, et al., 2017).

In both studies, the human and mouse microglia transcriptomes were compared. Gosselin et al. showed that human and mouse microglia are very similar, 13,253 of 15,768 orthologous genes pairs expressed within a fourfold range. At a cutoff of 10‐fold difference, they identified 400 human microglia enriched orthologous genes and 293 mouse microglia‐enriched orthologous genes. Human microglia are characterized by a higher expression of regulators of the complement system (*C2*, *C3*, *VSIG4*, *SERPING1*) and genes involved in brain structure development (*SYNDIG1*, *GLDN*, *CTTNBP2*, and *ROBO3*). Genes more highly expressed in mouse microglia included *Hexb*, *Sparc*, and *Sall3* (Gosselin et al., 2017).

In the study of Galatro et al., the human microglia transcriptome was also compared with mouse microglia transcriptomes (Grabert et al., 2016; Matcovitch‐Natan et al., 2016). An extensive overlap with human microglial data were observed, however, noteworthy differences were also observed. Human microglia‐specific genes were shown to involve in immune pathways, example genes are *GNLY*, *CD58*, *APOBEC3C*, *CLECL1*, *CD89*, and *CARD8* (Galatro, Holtman, et al., 2017). Most notably, when comparing age‐associated changes in microglia gene expression between humans and mice (Grabert et al., 2016), a suprisingly low overlap was detected. Most genes with an aging‐associated change in expression in humans were associated with the actin cytoskeleton (Galatro, Holtman, et al., 2017). Details on isolation methods, tissues used and which kind of comparison were used to identify microglia signature genes in these studies are summarized in Table 1.

**Table 1:** Mouse and human microglia transcriptomes identified by population sequencing

Study	Microglia isolation method	Tissue used	Sequencing method	Comparison	Signature genes	Representative genes
Gautier et al., 2012	Enzymatic dissociated with Liberase III, Percoll‐gradient separation, and fluorescence‐activated cell sorting (FACS) sorted as CD11b^+^CD45^lo^F4/80^lo^	Six week male mouse brain	Microarray	Compared with spleen red pulp macrophages (F4/80^hi^ B220^neg^ CD11c^hi^ MHC I^hi^); peritoneal macrophage (CD115^hi^ F4/80^hi^MHCII^neg^); lung macrophage (Siglec‐F^+^CD11c^+^MHC II^lo^). Genes upregulated in microglia by fivefold or more relative to their expression in the three other macrophage populations	65 genes	*Cx3Cr1*, *Siglech*, *Tmem119*, *Sall1*, *Hexb*
Chiu et al., 2013	Mechanically dissociation, Percoll gradient separation, CD11b^+^ magnetic beads purification	SOD1^G93A^, non‐Tg, SOD1^WT^ and lipopolysaccharides (LPS) injected mouse spinal cord	RNA‐seq	Compared with RNA‐seq data obtained from astroglia, motor neurons and whole spinal cord (fivefold increase; *q* < .05)	288 genes	*Olfm3*, *Tmem119*, *Siglech*, *Gal3st4*, *G530011006Rik*,*Csmd3*, *Slco2b1*
				Compared with microarray data from 22 other myeloid cell type collected by Immunological Genome Project (Gautier et al., 2012; fivefold increase, *q* < .05)	99 genes	
				The overlapping genes between above mentioned two data set	29 genes	
Hickman et al., 2013	Dissociated by Gentle Macs with enzymes (Dispase, collagenase III), Percoll gradient separation, and FACS sorted based on CD11b and CD45	5‐month‐old C57BL/6 mouse brain	Direct RNA‐seq	First identified 1,299 sensome genes. Next, compared with whole brain, top 100 sensome genes highly enriched in microglia based on E value were selected (E = CMMR[microglia]/CMMR[brain])	100 sensome genes	*P2ry12*, *P2ry13*, *Tmem119*, *Gpr34*, *Siglech*, *Cx3cr1*, *Trem2*
				Compared the top 10% of transcripts with the highest expression in microglia with those in peritoneal macrophages (CD11b ^+^CD45^+^)	626 genes uniquely in microglia	
				Among the 25 most highly expressed transcripts that were also uniquely expressed in microglia over macrophages (*p* < .00001, log2 fold change >4), microglia sensome gene were identified	Seven sensome genes out of 25 genes	
Butovsky et al., 2014	Single cell suspension, Percoll gradient separation, and FACS sorted based on CD11b^+^ CD45^lo^	C57BL6 mouse brain	Microarray	Compared with CD11b^+^Ly6C^+^ spleen‐derived monocytes	1,572 genes	*Fcrls*, *P2ry12*, *Mertk*, *Pros1*
			Mass spectrometry	Compared with CD11b^+^Ly6C^hi^ and CD11b^+^Ly6C^lo^ spleen‐derived monocytes (mass spectrometry; greater than twofold difference).	455 proteins (74 uniquely expressed in microglia	*P2ry12*, *Lgmn*, *Tppp*, *Bin1*, *Rgs10*
			Microarray were generated based on abovementioned mass spectrometry and array data	Compared with 10 types of immune cells, eight types of F4/80^+^ CD11b^+^ organ macrophages	239 genes	*Fcrls*, *C1qa*, *P2ry12*, *Pros1*, *Mertk*, *Gas6*
				Compared to other CNS cells (astrocytes, oligodendrocytes and neurons)	106 genes	*Fcrls*, *Olfml3*, *Tmem119*, *P2ry12*, *Hexb*, *Tgfbr1*
			Microarray contained 600 microRNAs	Compared to six types of organ macrophages and 11 types of immune cells	Eight microRNAs highly expressed	miR‐125b‐5p, miR‐342‐3p, miR‐99a
Bennett et al., 2016	Mechanical dissociation, myelin removal by beads, and FACS sorted based on Tmem119^+^ (E17 and part of P7 samples were based on CD45^lo^ CD11b + sorting)	E17, P7, P14, P21 and P60 mouse brain and LPS injected mouse brain	Paired‐end RNA‐seq	First identified top 100 microglia enriched genes over non‐microglia CNS myeloid cells (Tmem119^−^CD11b^+^ CD45^hi^) from P60 (>16‐fold). Next, tried to identify consistent microglia enriched genes during the development.	37 of 100 top microglia‐enriched genes are upregulated from E17 to P60	*Olfml3*, *Fcrls*, *Slc2a5*, *Ltc4s*, *Cx3Cr1*, *Selplg*, *P2ry12*, *Ccr5*, *Plxdc2*
Gosselin et al., 2017	Mechanical dissociation, Percoll gradient separation, and FACS sorted as CD11b^+^CD45^Lo^CD64^+^CX3CR1^Hi^ live cells, excluding most activated cells with moderate to high levels of CD45	19 human brain tissues resected for treatment of epilepsy, brain tumors, or acute ischemic	RNA‐seq	Microglial gene signature genes were identified with a cutoff of 10‐fold increased expression relative to cortex tissue (FDR < 0.05)	881 genes	*C3*, *CSF1R*, *SPP1*, *CX3CR1*, *P2RY12*, *C1QB*, *C1QA*
				First, differentially expressed genes list in neurodegenerative and behavioral disorders derived from microarray or RNA‐seq of intact tissue from 46 publicly available data sets were obtained. Second, overlapping genes between 881 microglia signature genes with those differentially regulated genes were identified.	For example, 97 microglia genes were positively correlated with Braak stage of Alzheimer's disease (AD) in prefrontal cortex tissue	*C1QA*, *C1QC*, *IRAK3*, *SPP1, SLC1A5, SLC7A7, TNFRSF10B, TNFRSF1B*
				Based on previous identified AD, Parkinson's disease (PD), multiple sclerosis (MS) and schizophrenia (Scz) risk alleles (Welter et al., 2014), many of these genes were preferentially expressed in microglia compared to cortex tissue	AD, for example, 28 of 48 AD genes was higher in microglia	*TREM2*, *SORL1*, *INPP5D*, *MEF2C*, *CD33*
Galatro, Holtman, et al., 2017, Galatro, Vainchtein, Brouwer, Boddeke, & Eggen, 2017	Mechanical dissociation, Percoll gradient separation, and FACS sorted as DAPI^neg^CD11b^high^CD45^int^ event	Post‐mortem right parietal cortex from donor without apparent neuropathological abnormalities	RNA‐seq	Microglial gene signature genes were identified with a cutoff log fold change >3 and adjusted *p* < .001 compared to cortex tissue	1,297 genes	*CX3CR1*, *CSFR1*, *C1QA‐C*, *CLECL1*, *CIITA*, *ITGAM*, *ICAM‐1*, *CD33*, *SIGLEC5/7‐12/14*
				Using age as a quantitative variable, microglia signature gene expression was examined in donors ranged between 34 and 102 years	212 genes increased and 360 genes decreased in expression during ageing	*ITGAL*, *TLN1*, *PFN1*, *VASP*, *P2RY12*, *IL6R*, *TLR10*, *ICAM3*, *ROBO2*, *SEMA3C*
Grabert et al., 2016	Mechanical dissociation, Percoll gradient separation, purified by anti‐CD11b microbeads	Cerebellum, cortex, hippocampus and striatum isolated from mice brain	Microarray	Genes differentially expressed by brain region (*p <* .05 with FDR correction).	2,527 genes	
				Gene coexpression analysis of the region‐specific microglial phenotypes by BioLayout express	Genes enriched in cerebellum microglia were immune‐related	*Clec4e*, *Clec7a*, *Stat1*, *Stat4*, *Irf7*, *Oasl1*, *H2‐D1*, *H2‐Aa*, *CD74*
					Genes enriched in cerebellum and hippocampus microglia related to energy production system	*Pfkp*, *Cat*, *Sod1*, *Sod2*, *Mdh1*, *Pparg*, *Ndufa1*, *Atp5a1*, *Cox5b*
Ayata et al., 2018	Translating ribosome affinity purification (TRAP). Mechanical dissociation, cell lysis, and RNA was purified by anti‐GFP beads	Microglia RNA isolated from cerebellum and striatum from TRAP mice.	Microglia‐specific TRAP‐sequencing; (also confirmed by single‐nuclei RNA‐seq)	Differential expression analysis by DESeq2 software	297 cerebellar microglia enriched genes (associated with cell clearance functions)	*Axl*, *H2‐Aa*, *Apoe*, *Mrc1*, *Cd74*, *Lyz2*, *Lilrb4*, *Colec12*
					733 striatum microglia enriched genes (related with mature microglia‐specific homeostatic surveillance)	*Slc2a5*, *Ecscr*, *Fscn1*, *Sall3*, *Arhgap30*, *Acap2*, *Fcrls*, *Mafb*, *Hhex*
Guneykaya et al., 2018	Mechanical dissociation, Percoll gradient separation, and purified by anti‐CD11b magnetic beads	Male and female mouse hippocampus and cortex	RNA‐seq	Genes differentially expressed between males and females in hippocampus and cortex (adjusted *p* < .01, log2 fold change >0.5 or <−0.5)	1,109 genes uniquely differentially expressed in hippocampus, 55 genes in hippocampus. 46 genes were differentially expressed in both region	*Irak1*, *Tmem 50b*, *Tmem33*, *Tmem30a*, *Atp11c*, *Atp6ap2*, *Kdm5d*
Van der Poel et al., 2019	Mechanically dissociation, enzymatic digestion with collagenase, Percoll gradient separation, negative selection with anti‐CD15 and positive selection with anti‐CD11b beads	Human brain cortical grey matter (GM), corpus callosum white matter (WM)	RNA‐seq	Differentially expressed genes between GM and WM in control and MS donors (fold change >2 or <−2, *p <* .05 with FDR correction)	453 genes in control, 124 genes in MS donors	GM enriched genes: *CCL2*, *TNFRSF25*; WM enriched genes: *CXCR4*, *ACKR1*, *GPNMB*, *NUPR1*

Abbreviations: AD, Alzheimer's disease; FACS, fluorescence‐activated cell sorting; GM, grey matter; LPS, lipopolysaccharides; MS, multiple sclerosis; PD, Parkinson's disease; Scz, Schizophrenia; TRAP, translating ribosome affinity purification; WM, white matter.

### 1.2 Mouse and human microglia heterogeneity revealed by bulk population sequencing

Microglia are plastic cells and their morphology, phenotype, and immune response display region‐dependent heterogeneity (Lawson, Perry, Dri, & Gordon, 1990; Yang et al., 2013). A detailed study of the basal ganglia region revealed region‐specific phenotypes of mouse microglia and this microglial diversity was partly determined by the local microenvironment (De Biase et al., 2017). At the transcriptional level, Grabert et al. was the first to demonstrate transcriptional differences between mouse microglia from the cerebral cortex, hippocampus, cerebellum, and striatum. Cerebellar and hippocampal microglia exhibited a more immune‐vigilant state, with higher expression of the genes *Camp* and *H2‐Ab1* (Grabert et al., 2016). Using a microglia‐specific translating ribosome affinity purification approach, it was determined that cerebellar mouse microglia displayed a more pronounced cell clearance phenotype (Ayata et al., 2018). In contrast, using bulk population RNA‐seq, Li et al. detected very limited transcriptomic heterogeneity between Tmem119^+^ FACS sorted microglia isolated from the above mentioned brain regions (Li et al., 2019).

Besides regional differences, gender‐dependent heterogeneity in microglia gene expression was also reported. Transcriptomic profiles were generated of microglia from male and female mouse hippocampus and cortex. Male mouse microglia displayed a higher capacity to present antigens and increased responsiveness to purinergic stimuli (Guneykaya et al., 2018). Expression profiling of microglia isolated from male and female mice revealed that the gene expression program in male microglia was delayed (Hanamsagar et al., 2017).

Microglia maturation during development is shaped by microbiome‐derived short chain fatty acids (Erny et al., 2015) and the effect of the microbiome on mouse microglia differentiation is sexually dimorphic (Thion et al., 2018). Perturbation of the microbiome had more profound effects in male embryos and female adults. These studies show that the microbiome is important for microglia development and maturation.

Regional heterogeneity was also demonstrated in the human CNS. Human microglia were isolated from grey matter (GM; occipital cortex) and white matter (WM; corpus callosum) from postmortem control and multiple sclerosis (MS) donor CNS. Between WM and GM microglia, 453 differentially expressed genes (logFC >2) were detected in samples from control donors, and 124 genes in MS donor‐derived samples. Genes highly expressed in control GM microglia were related to “cytokine‐mediated signaling,” such as *TNFRSF25* and *CCL2*; WM microglia were enriched for genes involved in “chemotaxis” and “inflammatory response” (*CXCR4*, *ACKR1*, *GPNMB*, *NUPR1*; van der Poel et al., 2019).

### 1.3 Single‐cell RNA‐sequencing (scRNA‐seq) of mouse and human microglia

Expression profiling of bulk population human microglia revealed changes associated with age, neurodegenerative diseases and psychiatric disorders (Galatro, Holtman, et al., 2017; Gosselin et al., 2017), and regional and gender‐dependent mouse microglia heterogeneity (Ayata et al., 2018; Grabert et al., 2016; Guneykaya et al., 2018). However, expression profiling of populations of cells in bulk precludes the identification and characterization of microglia subpopulations that (might) exist in the homeostatic brain or that evolve during CNS aging or disease.

ScRNA‐seq studies revealed spatio‐temporal diversity of mouse and human microglia gene expression during development and in an amyloid mouse model for Alzheimer's disease (AD). Multiple microglia subpopulations were identified that may contribute to, or at least change during, development and/or disease progression (Hammond et al., 2019; Keren‐Shaul et al., 2017; Masuda et al., 2019; Matcovitch‐Natan et al., 2016; Mathys et al., 2017).

The first disease‐associated single‐cell mouse microglia study was published by Keren‐Shaul et al. (Keren‐Shaul et al., 2017). In 5XFAD mice, an amyloid AD mouse model, a cluster of disease‐associated microglia (DAM) was identified, characterized by the upregulation of genes such as *Apoe*, *Trem2*, and *Tyrobp*. These genes are associated with lipid metabolism and phagocytosis and were already previously identified in a meta‐analysis of microglia gene expression changes in relation to aging and CNS disease (Holtman et al., 2015). These disease‐associated microglia subtypes might be promising targets for treatment of neurological diseases (Deczkowska et al., 2018). In these DAMs, genes associated with homeostatic microglia, such as *P2ry12* and *Tmem119*, were downregulated. While the scRNA‐seq study was performed in a mouse model for AD, the expression of some DAM signature genes was confirmed by immunostaining in human AD brain tissue, where these DAMs spatially associated with sites of AD pathology (Keren‐Shaul et al., 2017).

Hammond et al., analyzed microglia states during development, aging and injury in mice (Hammond et al., 2019). A number of distinct subpopulations of microglia were detected that peak in number during early development, expand during aging and emerge after injury. During mouse aging, small populations of interferon‐responive microglia appear. Additionally, several genes are almost uniquely expressed early during development, these genes include *Arg1*, *Rrm2*, *Ube2c*, *Cenpa*, *Fabp5*, *Spp1*, *Hmox1*, and *Ms4a7*. Importantly, the microglial diversity and the number of cells in each microglial subpopulation was not influenced by the sex of the mice. At P4/P5, a major microglial state was found, referred to as axon tract‐associated microglia (ATM). These microglia express genes such as *Spp1*, *Igf1*, *Gpnmb*, *Lgals1*, *Lgals3*, *Lamp1*, and *Cd68*. Using smFISH, Hammond et al., confirmed that these cells resided in the subcortical axon tracts of the corpus callosum in the forebrain and in the axonal tracts of the cerebellum. Interestingly, ATMs are concentrated at sites where myelination will occur, but are absent before myelination starts. To address changes in microglia during demyelination, mice were injected with lysolecthin (LPC). An injury‐responsive microglia cluster was identified where canonical microglia genes such as *P2ry12* and *Cx3cr1* were downregulated, and *Apoe*, *Ifitm3*, *Cst7*, *Axl*, and *Lpl* upregulated. These findings match the previously reported DAM phenotype (Keren‐Shaul et al., 2017).

Microglia development and demyelination at the single‐cell level were also studied by Masuda et al. (Masuda et al., 2019). Additionally, single‐cell RNA sequencing of 1,602 human microglia from healthy controls and MS patients was studied. Distinct clusters of microglia associated with MS pathology were identified. Genes upregulated in these clusters were *APOE*, *MAFB*, *CCL2*, *CCL4*, and *EGR2* whereas homeostatic genes such as *P2RY12* and *TMEM119* were downregulated.

### 1.4 Human microglia transcriptome revealed by single nucleus RNA‐seq

A major limitation of scRNA‐seq of human microglia is the requirement of fresh tissue to isolate viable cells from. Recently, single‐nucleus RNA‐sequencing (snRNA‐seq) was developed as an alternative technology which can successfully capture single cell transcriptomes from frozen tissues (Hu et al., 2017; Lake et al., 2016).

One of the first single‐nucleus RNA sequencing studies was performed by Lake et al. (Lake et al., 2016). From six young (<50 years) control donors, nuclei were isolated and subjected to droplet‐based single‐cell RNA sequencing, resulting in a total of 35,289 nuclei to characterize the cell types in the human brain. Thirty‐five distinct cellular clusters were defined, containing excitatory and inhibitory neurons, granule cells, Purkinje neurons, endothelial cells, pericytes, astrocytes, oligodendrocytes, oligodendrocyte precursor cells (OPCs), and microglia. Interestingly, an overrepresentation of neurons was observed, indicating a bias in sample processing or uneven detection rates for the different cell types with lower RNA content.

SnRNAseq was used to elucidate the transcriptomic changes underlying AD (Mathys et al., 2019). Mathys et al. isolated 80,660 nuclei from 48 individuals followed by droplet‐based single‐nucleus sequencing. 1,031 cell‐type specific gene expression changes were detected that were related to AD pathology. Early versus late stage AD pathology and female/male differences were analyzed, which were mainly restricted to neurons and oligodendrocytes/OPCs.

### 1.5 Databases for transcriptome information

There are several online databases that can be used to obtain information on quantitative expression analysis in microglia. The most recent applications are: GOAD (Holtman et al., 2015), Brain RNA‐Seq (Zhang et al., 2014), and Neuroexpresso (Mancarci et al., 2017). The aim of the GOAD database was to generate a platform for glia transcriptomics. Information from the most important glia transcriptome studies was aligned, quantified, and stored in a database. Brain RNA‐Seq (http://www.brainrnaseq.org/; Zhang et al., 2014) provides open access to a comprehensive mouse and human CNS cell types transcriptome data set. The interface of the web application is easy to use. In addition, it is possible to access quantitative expression datasets that are publicly available on the website. Neuroexpresso (http://neuroexpresso.org/; (Mancarci et al., 2017) is a database that combines data generated using GPL339 and GPL1261 micro‐array chips together with a single cell RNA‐Seq dataset (Tasic et al., 2016). Only glia samples after postnatal day 14, from wild‐type mice under control conditions are available. A single cell atlas of mouse microglia throughout the mouse lifespan and CNS injury is accessible at http://www.microgliasinglecell.com/ (Hammond et al., 2019). Following up on our previous database, GOAD we recently launched a new, improved glia transcriptome database BRAIN‐SAT, http://brainsat.eu/. It has features available on an interactive platform that allows access to recent, high quality bulk and single cell RNA‐Seq data of glial cells. Several functions are offered, including gene search, differential and quantitative expression analysis, and a single cell expression analysis feature that enables the exploration of published data sets at different levels.

### 1.6 Nuclear and whole‐cell transcriptomes

Some studies confirmed a high concordance between nuclear and whole‐cell transcriptomes in neurons (Bakken et al., 2018; Lake et al., 2017). However, the direct comparison between cellular and nuclear transcriptomes of microglia (and other glia) is yet lacking. Importantly, microglia are underrepresented in brain‐derived single nucleus RNA‐Seq data due to the relatively low abundance of microglia in these samples. Hence, it is important to enrich for microglia nuclei to determine subpopulations and changes therein at sufficient resolution. In addition, it is unclear how closely a frozen nuclear microglia transcriptome recapitulates the gene expression profile of freshly isolated microglia. Here, we generated matched nuclear and cellular microglia cell and nucleus RNA‐seq datasets to investigate whether nuclear transcriptomes are a good proxy for the cellular transcriptome.

## 2 MATERIALS AND METHODS

### 2.1 Animals

Male C57BL/6 mice (22–25 g; Envigo, the Netherlands) between 8 and 10 weeks of age were used for all experiments. Mice were raised on a 12‐hr light/dark cycle with food and water available ad libitum and were individually housed. All experiments were performed in the Central Animal Facility (CDP) of the UMCG, with protocol (15360‐03‐003) approved by the Animal Care and Use Committee (DEC) of the University of Groningen. Mice were given an intraperitoneal (ip) injection of 1 mg/kg lipopolysaccharide (LPS) (Sigma‐Aldrich, *Escherichia coli* 011:B4,L4391, Saint Louis, MD, USA) dissolved in DPBS (Lonza, BE17512F, Walkersville, MD, USA). Control mice received a respective volume of DPBS. After 3 hr, animals were sacrificed under anesthesia and the brain was collected.

### 2.2 Microglia and nuclei isolation

#### 2.2.1 Microglia isolation from mouse and human brain tissue

Microglia were isolated from adult mouse brain using the protocol as described before (Galatro, Vainchtein, et al., 2017). Briefly, the brains were isolated and triturated using a tissue homogenizer. The homogenized brain samples were passed through a 70 μM cell strainer to obtain a single cell suspension. The cells were centrifuged at 220 rcf for 10 min at 4°C and the pellet was resuspended in 24% Percoll gradient buffer. 3 mL dPBS was pipetted onto the gradient buffer and myelin was removed by centrifuging at 950 rcf for 20 min at 4°C. The cell pellets were incubated with the antibodies CD11b‐PE (clone M1/70, eBiosciences, San Diego, CA, USA), CD45‐FITC (clone 30‐F11, eBiosciences), and Ly‐6C‐APC (clone HK1.4, BioLegend, San Diego, CA, USA). Microglia were FACS sorted as DAPI^neg^ CD11b^high^ CD45^int^ Ly‐6C^neg^ events. For each condition, microglia from three mice were combined into one lane of a 10X Genomics Chromium chip. Human samples were loaded individually.

Post‐mortem human brain tissue of the superior frontal gyrus of two donors was obtained from the Dutch Brain Bank. Microglia were FACS sorted as DAPI^neg^DRAG5^pos^CD45^pos^CD11B^pos^, as previously described (Galatro, Holtman, et al., 2017). For bulk sequencing, three mice were used per condition and sequenced separately. For single cell/nucleus sequencing, sorted microglia cells/nuclei from three mice/condition were pooled and loaded on a 10× Genomics Chromium chip according to the manufacturer's instructions.

#### 2.2.2 Nuclei isolation from sorted microglia

The nuclei isolation protocol was adopted from (Krishnaswami et al., 2016). After FACS isolation, microglia were pelleted by centrifugation at 600× *g* for 10 min, 4°C. Cells were resuspended in 400 μl cold homogenization buffer (NIM2 with 0.1% Triton X‐100 and 0.4 U/μl RNasIn). Cells were gently vortexed for 10 s and incubated on ice for 10 min. Nuclei were pelleted by centrifugation at 1,200*g* for 8 min, 4°C, resuspended in 200 μl resuspension buffer (NIM2 with 0.4 U/μl RNasIn) and transferred to a FACS tube. Another 200 μl resuspension buffer was used to wash the tube, to recover all nuclei. Before sorting, DAPI was added, nuclei were FACS sorted as singlets, DAPI^pos^, CD11b^neg^, and CD45^neg^ events. For each treatment, microglia from three mice were combined into one lane of a 10× Genomics Chromium chip. Human samples were loaded individually.

#### 2.2.3 Nuclei isolation from frozen brain tissue

Nuclei were isolated as described previously with a few adaptations (van den Bos et al., 2016). Briefly, the tissue was cut in 40 μm sections on a cryostat and homogenized in a sucrose lysis buffer (10 mM Tris–HCL [pH 8.0]; 320 mM sucrose; 5 mM CaCl_2_; 3 mM Mg(Ac)_2_; 0.1 mM EDTA; 1 mM dithiothreitol [DTT] and 0.1% Triton X‐100). Around 15 sections of 40 μm, 1 cm^2^, per tissue sample were collected and lysed. The samples were filtered through a 70 μm cell strainer. Nuclei were purified by ultracentrifugation (107,000× *g* for 1.5 hr) through a dense sucrose buffer (10 mM Tris–HCL [pH 8.0]; 1.8 M sucrose; 3 mM Mg(Ac)_2_; 0.1 mM EDTA and 1 mM DTT). The supernatant was removed and the pellet was resuspended in 2% BSA/PBS. Samples were kept on ice throughout the isolation procedure. The nuclei were stained with antibodies NeuN‐AF647 (clone A60, Merck Millipore, Billerica, MA, USA), OLIG2‐AF488 (clone211F1.1, Merck Millipore) and DAPI. DAPI^pos^ NeuN^neg^ OLIG2^neg^ nuclei were FACS sorted and loaded on a 10× Genomics Chromium chip.

### 2.3 Library preparation

For bulk sequencing, total RNA was isolated from either sorted microglia or nuclei using the RNeasy Plus Micro Kit (Qiagen,74034). RNA quantity and quality were analyzed using an Experion electrophoresis system (Bio‐Rad Laboratories, Hercules, CA, USA). Sequencing libraries were prepared with the Quant Seq 3' mRNA‐Seq Library Prep Kit FWD (Lexogen, Vienna, Austria).

The single‐cell barcoded libraries were constructed using Single Cell 3' Reagent Kits v2 (10× Genomics). In brief, after sorting, single cells were partitioned into nanoliter‐scale Gel Bead‐in‐emulsions (GEMs) in the chromium controller. GEMs were then incubated in a thermal cycler to generate barcoded cDNA. After amplification, cDNAs were further processed for sequencing by ligation of adapters and individual sample indices. The libraries were sequenced on a NextSeq platform.

### 2.4 RNA‐sequencing data analysis

#### 2.4.1 Bulk sequencing analysis

Quality control of the raw FASTQ files was performed with FASTQC. Bad quality bases were trimmed with TrimGalore version 0.4.5. Sequences were aligned using HiSat2 version 2.1 to the *Mus musculus* (GRCm38.91) reference template obtained from Ensembl and quantified with featureCounts against the standard (spliced) reference genome, for both cells and nuclei. A quality check of aligned data was performed with FASTQC and MultiQC. Raw count matrices were loaded in R and annotated by converting the ensemble IDs to gene symbols using the corresponding gtf file. Only genes with >1 counts in at least two samples were included in the analysis. To determine whether mice from the same group would cluster together, the count matrix was normalized with the blinded variance‐stabilizing method from DESeq2 from Bioconductor and mitochondrial genes were removed prior to this analysis. Differential gene expression analysis was performed with the edgeR package from Bioconductor. Several comparisons were made, for all we used an absolute log fold change >1 and an FDR‐adjusted *p*‐value <.05. These results are plotted in heatmaps. Enrichment for GO terms for individual comparisons was performed by the EnrichGO function from clusterProfiler from Bioconductor. We used a *p*‐value and *q*‐value cutoff of .01.

#### 2.4.2 Single‐cell RNA‐sequencing analysis

Demultiplexed FASTQ files were used as input for the 10× Genomics pipeline Cell ranger (v3.0). Unspliced pre‐mRNA transcripts were counted according to the method described by 10x Genomics. Barcode filtering was performed with the package DropletUtils from Bioconductor. Genes that were expressed in >2 cells were used for further analysis. Bad quality cells/nuclei were removed based on >5% MT content. Duplicates were removed by setting an upper UMI threshold that was based on the multiplet rate as mentioned in the 10x genomics user guide. Samples were combined using the merge function and raw counts were normalized with the CRAN package Seurat (v3). For each cell, the counts of each gene were divided by the total sum of counts per cell. Then the counts were multiplied by a scale factor of 10,000 and log‐transformed. Highly variable genes were determined with the mean.var.plot function in R. With the ScaleData function, heterogeneity associated with mitochondrial content and ribosomal content was regressed out. Additionally, in order to prevent clustering based on differences in UMI count between cells and nuclei, the number of UMIs per cell/nucleus was corrected for. Principal component analysis was performed with default settings. Clustering was performed with Seurat and visualized in Uniform Manifold Approximation and Projection plots (UMAP). Visualizations were made with the CRAN package ggplot. Differential gene expression analyses were performed using MAST.

## 3 RESULTS

### 3.1 Comparison of nuclear and whole‐cell transcriptome by bulk sequencing

**Figure 1:**
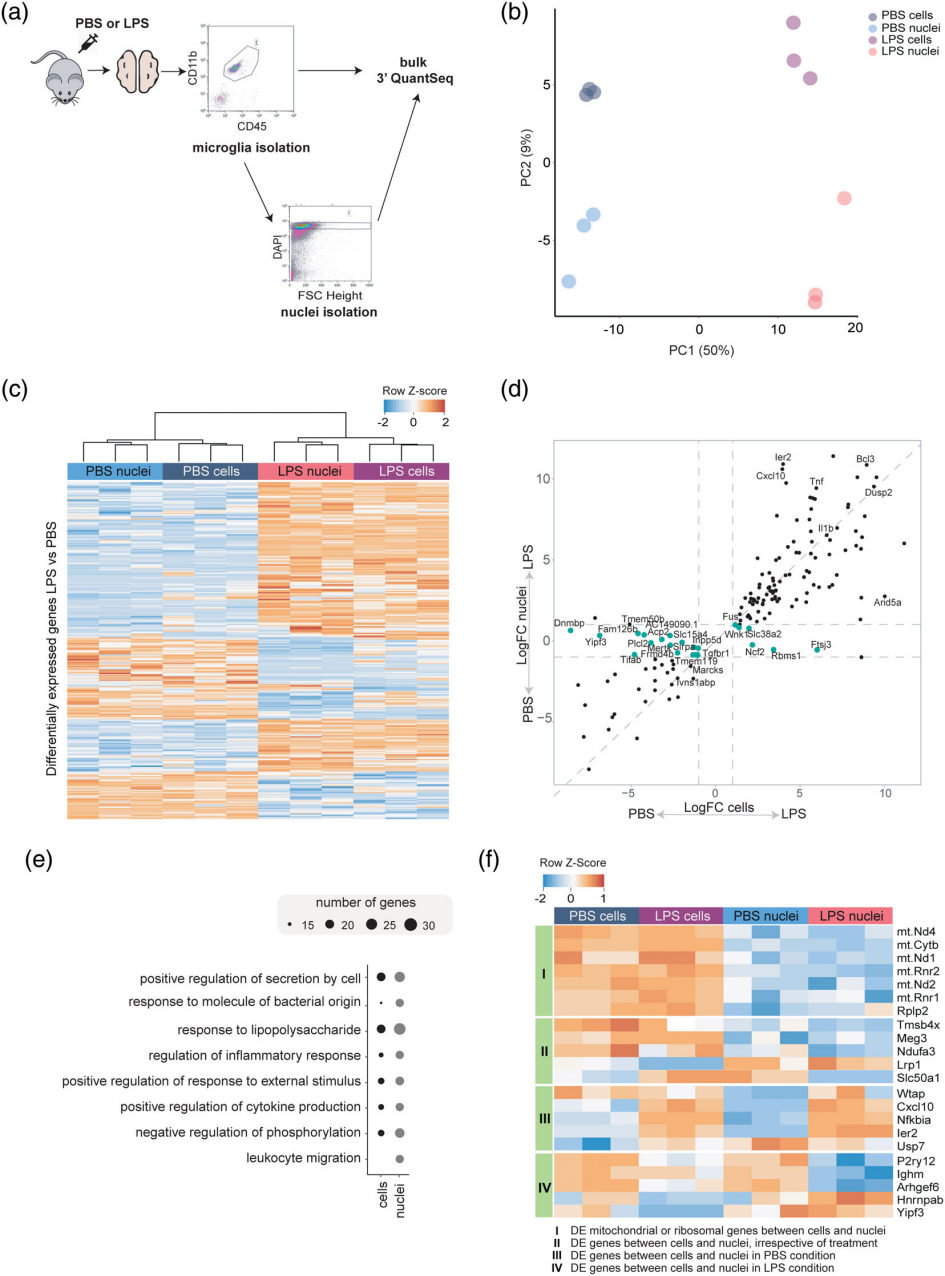
Microglia nuclear transcriptomes are a reliable proxy for cellular gene expression profiles in mice. (a) Experimental design. Mice received an ip injection with PBS or LPS (1 mg/kg; three mice per group) and after 3 hr, the animals were terminated. Microglia were isolated by FACS as CD11b^pos^CD45^int^Ly6C^neg^. From a part of the isolated microglia, nuclei were sorted as DAPI^pos^CD45^neg^ CD11b^neg^ events. After RNA isolation, the cellular and nuclear RNA was expression profiled using 3′ Quantseq (Lexogen). (b) Principal component analysis (PCA) of the transcriptomes across different groups. (c) Heatmap depicting LPS‐responsive genes (297 genes) in cells and nuclei (*n* = 3 mice). The colors indicate row‐*z*‐scores. Both rows and columns were ordered by unsupervised clustering. (d) Four way plot depicting genes significantly differentially expressed (logFC >1 and FDR < 0.05) between cells and nuclei from PBS or LPS‐injected mice. The X‐axis depicts the logFC in cells, the y‐axis the logFC in nuclei. Genes indicated in cyan have a logFC <1 in the PBS/LPS nuclei comparison. (e) GO analysis of LPS‐induced genes in cells and nuclei. The top eight most significant GO terms, associated with LPS‐upregulated genes in cells and nuclei are shown. The size of the circle indicates the number of genes associated with the respective GO term. (f) Heatmap of the 22 genes differentially expressed between cells/nuclei and PBS/LPS conditions. DE, differentially expressed

To evaluate whether nuclear microglia transcriptomes resemble cellular transcriptomes, we performed bulk RNA sequencing on sorted microglia and nuclei isolated from these microglia. Additionally, we included an LPS stimulus to determine whether an acute microglial response is conserved at the nuclear level. Microglia were isolated from mice, 3 hr after an ip injection with PBS or LPS; and from half of these microglia, nuclei were isolated. Sorted microglia and nuclei were expression profiled, with three biological replicates per group (cells/nuclei and PBS/LPS; Figure 1a). We detected 10,651 uniquely expressed genes in the PBS‐cells samples, 10,315 in the LPS‐cells samples, 10,225 in the PBS‐nuclei samples (88.3% overlap with PBS‐cells), and 9,336 in the LPS‐nuclei samples (82.8% overlap with LPS‐cells). Principal component analysis on these genes indicated segregation of the samples into four distinct groups, associated with PBS/LPS treatment (PC1) and cells/nuclei (PC2; Figure 1b), respectively. Differential gene expression analysis between the LPS and PBS samples showed that cells and nuclei had a highly similar transcriptional response to a peripheral LPS stimulus (158 DE genes in cells, 232 DE genes in nuclei; Table S1). Among these genes, in response to LPS, 111 genes were upregulated and 47 genes downregulated in cells, and 153 genes upregulated and 79 genes downregulated in nuclei (Figure 1c and Table S1). Importantly, when comparing the logFC values of the genes significantly differentially expressed in the LPS cells versus PBS cells, most of these genes had similar logFC values in the nuclei PBS‐LPS comparison, for example, *Cxcl10*, *Tnf*, and *Il1b*, indicating cells and nuclei respond very similarly to LPS (Figure 1d). However, 23 genes in the nuclei comparison had a logFC value <1, indicated by cyan dots in Figure 1d. Transcriptional changes in these genes in response to LPS are less pronounced in nuclei. We performed GO analysis to determine the functional properties associated with LPS responsive genes in cells and nuclei (Table S2). Only the top eight most significant terms are depicted, as these were most representative for the overall outcome. As expected, many significantly enriched terms were associated with the inflammatory response of microglia, and showed extensive overlap between cells and nuclei (Figure 1e).

To determine the similarity between cellular and nuclear microglia expression profiles, we performed differential gene expression analysis between cells and nuclei in both the PBS and LPS condition. Only 22 genes were differentially expressed between cells and nuclei after either PBS (12 DE genes) or LPS treatment (16 DE genes; Figure 1f). Seven of these differentially expressed genes (*mt‐Nd4*, *mt‐Cytb*, *mt‐Nd1*, *mt‐Rnr2*, *mt‐Nd2*, *mt‐Rnr1*, *Rplp2*) were mitochondrial‐ or ribosomal‐related and less abundant in nuclei. Five genes were differentially expressed between cells and nuclei, irrespective of treatment (*Tmsb4x*, *Meg3*, *Ndufa3*, *Lrp1*, *Slc50a1*). Five genes were differentially expressed between cells and nuclei after an LPS stimulus (*P2ry12*, *Ighm*, *Arhgef6*, *Hnrnpab*, *Yipf3*) and five genes were differentially expressed between the PBS samples but not after an LPS stimulus (*Wtap*, *Cxcl10*, *Nfkbia*, *Ier2*, *Usp7*). A detailed gene list is provided in Table S3.

In summary, these data indicate that mouse microglia nuclear transcriptomes are a close approximation of cellular transcriptomes when analyzed using bulk sequencing and that the transcriptional response to LPS observed in nuclei and microglia was highly similar.

### 3.2 Comparison of single microglia nuclear and cell transcriptomes

**Figure 2:**
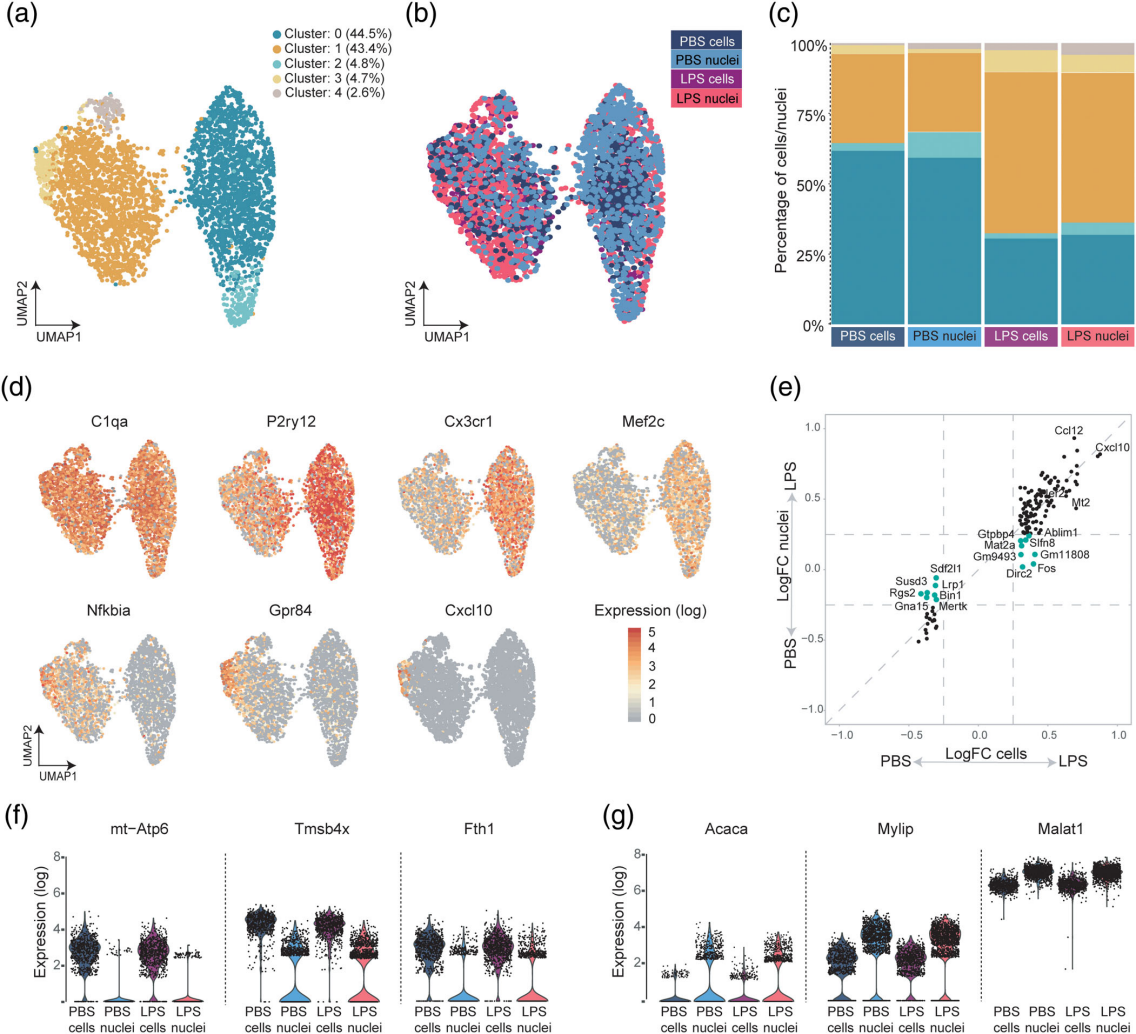
Single cell and nucleus RNA sequencing profiles of mouse microglia are highly similar. (a) UMAP plot with five clusters identified in the merged single microglia cell and nucleus transcriptomes from PBS‐ and LPS‐treated mice. Cells and nuclei from three mice were pooled and loaded on 10× chips. (b) UMAP plot where colors indicate the different experimental samples: microglia and nuclei from PBS‐ and LPS‐treated animals. (c) The distribution of clusters across the indicated experimental groups. (d) UMAP plot depicting expression (log‐transformed UMI counts per 10,000 transcripts) of canonical microglia gene *C1qa*, homeostatic genes *P2ry12*, *Cx3cr1*, and *Mef2c*, and LPS responsive genes *Nfkbia*, *Cxcl10*, and *Gpr84*. (e) A four way plot depicting genes significantly differentially expressed between cells and nuclei from PBS or LPS‐injected mice (average logFC <0.25 and adjusted *p* value <.01). The X‐axis depicts the logFC in cells, the y‐axis the logFC in nuclei. Genes indicated in cyan color have a logFC <0.25 in the in the PBS/LPS nuclei comparison. (f) Violin plots depicting distributions of normalized relative expression levels of cell‐enriched and (g) nucleus‐enriched genes

We next performed single mouse microglia cell‐ and nucleus‐sequencing to determine the overlap in their transcriptomes and the preservation of microglia heterogeneity. After filtering, 708 PBS cells, 1073 PBS nuclei, 802 LPS cells, and 1374 LPS nuclei were used for downstream analyses. Overall, we detected more uniquely expressed genes in cells than in nuclei (median gene number: PBS‐cells: 1326; PBS‐nuclei: 480; LPS‐cells: 1481; LPS‐nuclei: 470). Consistent with previous findings, our nuclear data showed a higher proportion of reads mapping to intronic regions and a lower percentage of mitochondrial genes (Figure S1a and S1b) (Bakken et al., 2018; Lake et al., 2017). In addition, a lower percentage of ribosomal genes was detected in the nuclei (Figure S1c). PCA analysis of the transcriptomes of individual cells and nuclei showed an extensive overlap, indicating that cellular and nuclear transcriptomes are quite similar (Figure S2a). To identify microglia subpopulations, the cells and nuclei data were combined for dimensionality reduction through UMAP and five clusters were identified using clustering analysis (Figure 2a). Since quality is an important variable that can have impact on clustering analysis, UMI count and unique gene count per cell were investigated and no clustering based on these parameters was observed (Figure S2b and 2c). Clusters 0 and 2 primarily consisted of PBS cells and nuclei and Clusters 1, 3, and 4 mainly contained cells and nuclei isolated from LPS‐treated mice (Figure 2b). Also, a large overlap between cells and nuclei was observed both after PBS and LPS stimulation (Figure 2b). Upon LPS stimulation, microglia shift from a homeostatic to an activated state resulting in an activated microglia subpopulation (Sousa et al., 2018). To confirm the induction of a subset of activated microglia, we determined the expression of homeostatic and activated microglia marker genes reported previously (Keren‐Shaul et al., 2017; Sousa et al., 2018). *C1qa* expression was detected in all cells and nuclei. Clusters 0 and 2 expressed high levels of homeostatic microglia marker genes such as *P2ry12*, *Cx3cr1*, and *Mef2c*. Clusters 1, 3, and 4 contained activated microglia with an increased expression of *Nfkbia*, *Gpr84*, and *Cxcl10* (Figure 2d). Distribution of clusters across different groups showed that the PBS cells and nuclei primarily consisted of Clusters 0 and 2 type microglia/nuclei; upon LPS stimulation, Clusters 1, 3, and 4 were increased in both LPS cells and nuclei, reflecting microglia activation (Figure 2c). These observations corroborated our bulk sequencing data, showing single microglia nucleus and cellular transcriptomes were highly similar, as well as the detected gene expression changes induced by LPS. In order to determine whether the LPS response was conserved in nuclei, LPS‐induced changes in gene expression in cells and nuclei were compared (Figure 2e). When comparing the logFC values of LPS nuclei versus PBS nuclei with LPS cells versus PBS cells, the majority of the genes responded similarly to LPS in cells and nuclei, for example, *Ccl12*, *Cxcl10*, and *Ler2* (Figure 2e). However, 15 genes had an average absolute logFC value <0.25 in LPS versus PBS nuclei (cyan dots in Figure 2e), indicating that LPS‐induced changes in the expression of these genes was less pronounced in nuclei. A detailed gene list of the PBS/LPS comparisons in cells and nuclei is provided in Table S4. Next, we performed differential gene expression analysis between cells and nuclei from PBS and LPS mice in the single cell/nucleus dataset. In the PBS samples, seven genes were enriched in cells compared to nuclei (logFC >1.5, FDR < 0.05), and except for *Tmsb4x*, these genes were all mitochondrial‐related (*mt‐Atp6*, *mt‐Cytb*, *mt‐Co3*, *mt‐Nd1*, *mt‐Nd4*, *mt‐Co2*) (Table S5). In the LPS samples, apart from the previous seven genes, five additional cell‐enriched genes were detected (*mt‐Co1*, *Rps23*, *Rps14*, *Fth1*, *Rpl32*; Table S5). Nucleus‐enriched genes were only detected in the PBS condition (*Acaca*, *Spag5*, and *Gm17660*) and with a less stringent cut off (logFC <−1 and FDR < 0.05), six genes were enriched in LPS nuclei (*Acaca*, *Kazn*, *Gm17660*, *Gm26916*, *Mylip*, *and Vps13a*) (Table S5). *Malat1* was more enriched in nuclei (logFC = −0.72 and FDR = 0 [PBS condition], logFC = −0.66 and FDR = 0 [LPS condition; Table S5), in agreement with earlier findings (Bahar Halpern et al., 2015; Bakken et al., 2018). Some representative genes, mentioned above, are depicted in Figure 2f,g. Taken together, single‐microglia nucleus gene expression profiles are a reliable proxy for single microglia transcriptomes in mice.

### 3.3 Comparison of nuclear and whole cell transcriptome by single cell/nucleus sequencing in human microglia

**Figure 3:**
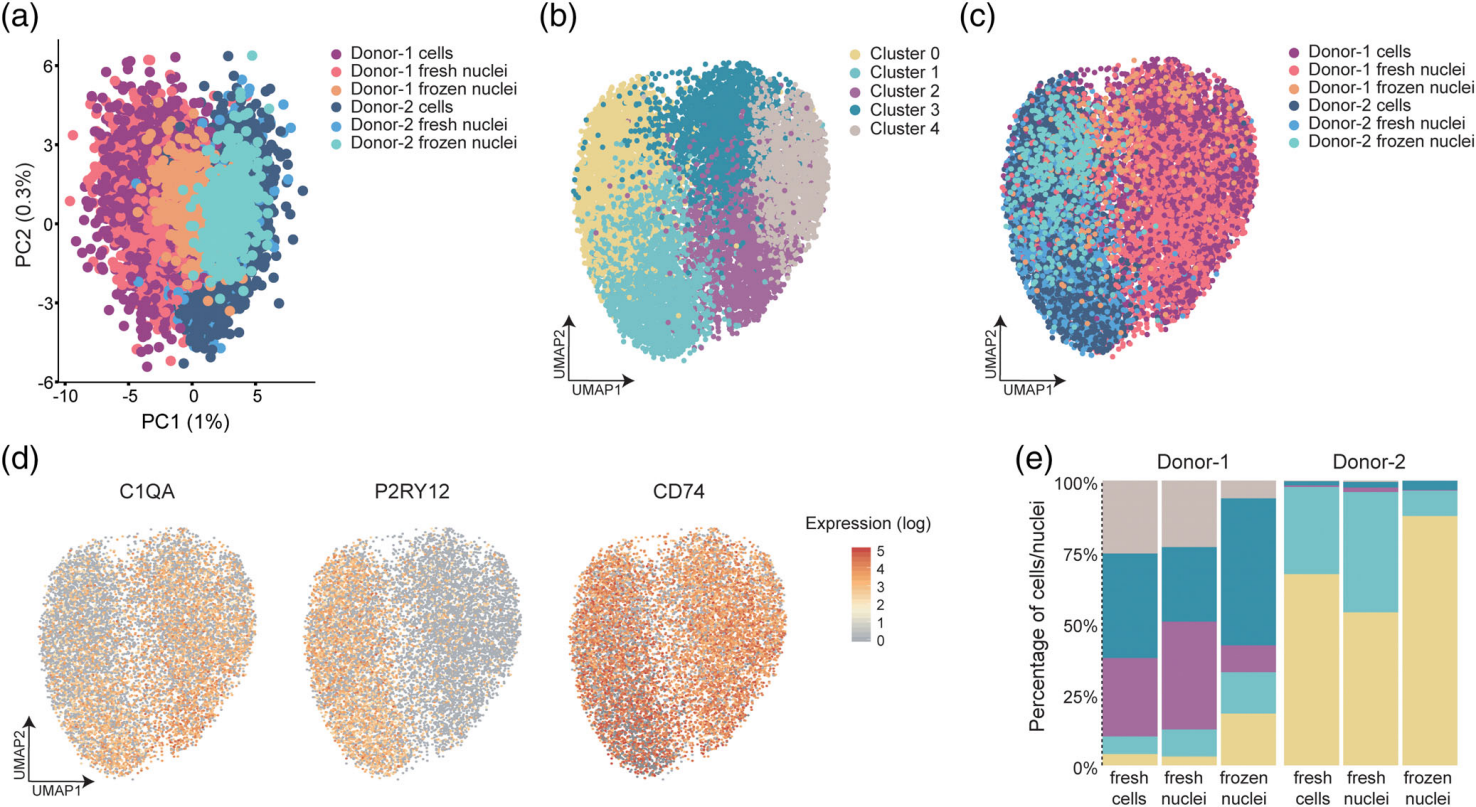
Single cell and nucleus RNA sequencing of human CNS tissues indicates that both fresh and frozen nuclear transcriptomes closely approximate and reflect microglia gene expression heterogeneity. (a) PCA plot of fresh‐tissue derived cells and nuclei, and nuclei isolated from adjacent frozen tissue samples. (b) UMAP plot depicting five clusters identified in the merged single cell and nucleus transcriptomes of microglia from two human donors. (c) UMAP plot where colors indicate the different experimental samples, fresh tissue‐derived microglia cells and fresh and frozen tissue‐derived nuclei. (d) UMAP plot depicting expression (log‐transformed UMI counts per 10,000 transcripts) of canonical microglial genes *C1AQ*, *P2RY12*, and *CD74*. (e) The proportion of clusters across the indicated experimental samples

To investigate whether a similar overlap between the cellular and nuclear transcriptomes was present in human microglia, we isolated microglia and microglia nuclei from fresh post‐mortem brain tissue of two human donors. In addition, we froze adjacent tissue blocks of the same donors and then isolated nuclei from these samples to evaluate whether nuclei isolated from frozen tissue can be used to determine microglia transcriptomes. After removal of doublets and low‐quality cells and nuclei, 2,620 cells, 3,836 fresh nuclei, 275 frozen microglia nuclei were obtained from Donor 1 and 2,653 cells, 2046 fresh nuclei, and 405 frozen microglia nuclei from Donor 2. Frozen microglia nuclei were obtained by exclusion of non‐microglia nuclei (like astrocytes) from the NeuN^neg^OLIG2^neg^ nuclei population. We observed a similar pattern in the distribution of mapping stats as we found in mouse, where intronic reads were more abundant in the nuclear samples and lower percentage of mitochondrial and ribosomal genes were detected in nuclei samples (Figure S1). PCA analysis of the transcriptomes of microglia cells and nuclei indicated that cells and nuclei overlap and that the variation in the dataset is mainly explained by the difference between the donors (Figure 3a). After dimensionality reduction and clustering, five microglia subclusters were identified (Figure 3b). Like in the mouse data, clustering was not affected by the quality of the cells/nuclei (Figure S3a and 3b). Clear donor variation was observed in the UMAP visualization, where Clusters 0 and 1 were mainly derived from Donor 2, and Clusters 2, 3, and 4 were primarily derived from Donor 1 (Figure 3b,c). The microglia gene *C1QA* was more abundant in microglia from Donor 1, where homeostatic gene *P2RY12* was more abundantly expressed in microglia from Donor 2. *CD74* was more homogeneously expressed across microglia from Donors 1 and 2 (Figure 3d). The distribution of different clusters across different groups showed that, similar to the mouse data, freshly isolated nuclei from human microglia cells are a good proxy for the human single cell microglia transcriptome (Figure 3e). Importantly, microglia nuclei isolated from frozen CNS tissue contained all the subpopulations identified in the fresh nuclei/microglia samples from the same donors but with a small shift in cluster ratios (Figure 3e). Nuclear and cellular transcriptomes were generated from the same FACS‐sorted microglia sample, allowing for a direct comparison between the fresh cells and nuclei, within each donor. However, for the frozen nuclei, although from the same donors, nuclei were isolated from adjacent tissue using a different isolation method, tissue homogenization, and sucrose density centrifugation. The modest difference in cluster composition in the frozen nuclei might be caused by differences in the isolation protocols for fresh and frozen nuclei, and that slightly different areas of brain tissue were used for the fresh and frozen nuclei isolation, with potential differences in WM/GM composition leading to altered cluster composition (van der Poel et al., 2019). We detected 30 differentially expressed genes (abs[logFC] > 0.5, adjusted *p* value <.01) between cells and fresh nuclei in Donor 1, of which 19 were ribosomal or mitochondrial genes. The other genes were *LINC00486*, *CEBPD*, *MALAT1*, *PNISR*, *TPT1*, *NBEAL1*, *BAIAP2L1*, *DHFR*, *MPHOSPH8*, *TIAM2*, and *NGB* (Table S6). Between cells and fresh nuclei from Donor 2, 75 genes differentially expressed, of which 46 were ribosomal or mitochondrial. The other genes were *AC018541*, *RBFOX1*, *TPT1*, *AC120193*, *MELK*, *OOEP*, *NBEAL1*, *PLEKHA7*, *TMSB10*, *PNISR*, *APOO*, *TMSB4X*, *PLEKHA6*, *AIF1*, *CDH18*, *FCER1G*, *BAIAP2L1*, *ATP5F1E*, *PFDN5*, *SIK3*, *DAPK1*, *UQCRB*, *UBA52*, *COMMD6*, *ACO024230*, *MAP3K15*, *MECOM*, *LINC00871*, and *DHFR* (Table S6).

Overall, microglia nuclear transcriptomes from both fresh and frozen CNS tissue are a good proxy for freshly isolated microglia and potential subpopulations present in the tissue.

## 4 DISCUSSION

Single nucleus RNA sequencing is considered to have several advantages over single cell RNA sequencing. First, nuclei are more resistant to mechanical stress and cryopreservation, which would make large collections of well‐characterized (frozen) tissues in biorepositories amenable for single nucleus profiling (Krishnaswami et al., 2016). Second, single‐nucleus RNA sequencing is less cell type‐biased than single cell RNA sequencing. Some cell (sub)types are more vulnerable to tissue dissociation than other cell types, resulting in potential under‐ and over‐representation of cell types or subsets thereof in the data (Bakken et al., 2018). Several studies using brain tissue have shown that nuclei reflect transcriptional changes at the tissue level, single cell level and also recapitulate subtypes and diversity in neurons (Habib et al., 2016; Lake et al., 2016; Lake et al., 2017). However, for microglia, it is yet unknown whether nuclei can serve as an alternative for cellular transcriptomes, whether nuclear transcriptomes contain enough information to identify microglia subtypes, and whether this is amenable to frozen CNS tissues. Hence, we performed a systematic comparison of mouse and human fresh microglia and nuclei, and additionally microglia nuclei isolated from frozen human brain tissue.

The number of microglia in the brain is relatively low (Keller, Ero, & Markram, 2018) and, as a consequence the number of microglia in total CNS tissue single cell sequencing data is also relatively low (Darmanis et al., 2015; Mathys et al., 2019). In order to enrich for microglia nuclei from frozen tissue, nuclei from neurons and oligodendrocytes were labeled with antibodies against NeuN and OLIG2 and selected against during FACS isolations. After sequencing, first the NeuN/OLIG2 double negative nuclei population was clustered, and the microglia nuclei population was extracted. Microglia nuclei were identified based on a.o. *HEXB* expression where markers for astrocytes (*AQP4*), neurons (*MAP1B*), and oligodendrocytes (*PLP1*) were not detected in mouse (Figure S2d) and human (Figure S3c) microglia nuclei.

To investigate the differences and similarities between single cell and single nucleus RNA sequencing (Selewa et al., 2019), we compared these technologies in LPS‐challenged mice, as that induces a strong and well characterized transcriptional response in microglia (Holtman, Raj, et al., 2015). First, we used bulk RNA sequencing to determine to what extent the nuclear and cellular gene expression profiles in mice overlap. As expected, we obtained less RNA from nuclei, approximately 20% of the amount of total RNA typically isolated from microglia (data not shown). Only 22 genes were differentially expressed between mouse cells and nuclei, indicating a highly similar expression profile (Figure 1f). Additionally, the consistent absence of mitochondrial and ribosomal genes in the nuclei samples indicated the successful isolation of pure nuclei and no contamination with ambient cellular RNA. Using a cut off LogFC >1, FDR < 0.05, in cells 158 differentially expressed genes were detected and 232 genes in nuclei, indicating that the transcriptional changes induced by LPS are more pronounced in the nuclear transcriptome. This may be explained by the fact that nuclei primarily contain newly generated transcripts, hence reflecting active transcription whereas a cellular transcriptome consists of already present plus newly formed transcripts. Although less sensitive than bulk sequencing, single‐cell sequencing can detect cellular heterogeneity, which is often masked by bulk sequencing. Next, we investigated whether single nucleus sequencing could recapitulate single cell sequencing and whether microglia subtypes could still be detected. Since very limited transcriptomic heterogeneity was observed in adult homeostatic mouse microglia (Li et al., 2019), we decided to include an LPS challenge. Three hours after an ip LPS injection, a subset of microglia had shifted from a homeostatic (Clusters 0 and 2) to an activated state (Clusters 1, 3, and 4) (Figure 2c,d), which was different from the transcriptional shift observed in microglia 24 hr after an LPS challenge where all cells lost their homeostatic signature and were activated (Sousa et al., 2018). Importantly, the cluster distribution of the cells and nuclei in both treatment groups was very similar, indicating that the heterogeneity observed in the cells and nuclei is rather similar, also during activation. Direct comparison of the LPS response in cells and nuclei (both using bulk and single‐cell data), indicated that most of the LPS‐induced transcriptional changes in microglia were also detected in nuclei (Figures 1d and 2e).

Next, we investigated whether human microglia nuclei, including nuclei isolated from frozen CNS tissues, could reliably recapitulate cellular transcriptomes generated with microglia isolated from fresh CNS tissue. Clustering analysis of cells and nuclei from both donors combined showed that cells and fresh nuclei clustered very similarly. The distribution of clusters in frozen nuclei was slightly altered but all the clusters detected in fresh microglia were recapitulated in frozen nuclei (Figure 3e). The fresh nuclei were isolated from the same microglia sample used for cellular profiling, and hence should be extremely similar. The frozen nuclei were isolated from an adjacent tissue block of the same donor which may had a slightly different cellular composition. Differences in the relative amounts of WM and GM between the fresh and frozen tissue samples would already result in changes in gene expression and cluster sizes. In addition, the isolation methods used for fresh and frozen nuclei were different, possibly contributing to the observed differences by preferential enrichment or loss of nuclear subtypes (Cluster 2 in Donor‐1 and Cluster 1 in Donor‐2), due to different sensitivities to freeze–thaw attrition. Importantly, donor variation, a reported parameter in single‐cell microglia data (Olah et al., 2018), was equally detected in microglia cells and both fresh and frozen nuclei in the two donors analyzed. This indicates that donor‐associated changes in gene expression were preserved in frozen microglia nuclei and that they hence reliably recapitulate the gene expression profile of fresh tissue microglia (Figure 3e).

By comparing nuclear and whole cell microglia transcriptomes by bulk sequencing and single nucleus/cell sequencing in human and mouse, we confirm that microglia nuclei are a reliable proxy for the single cell microglia transcriptome. This enables the use of banked human specimens to investigate microglia in neurodegenerative disease and neurological disorders.

## 5 FUTURE PERSPECTIVES

Gene expression profiling of isolated cells, either in bulk or at the single cell levels, is inherently associated with loss of spatial, contextual information of the used tissue. Relatively recently, two technologies were reported where gene expression profiles were generated while retaining spatial information of the analyzed tissue section. The first technology, spatial transcriptomics (Stahl et al., 2016), makes use of glass slides on which oligo d(T) primers with positional barcodes are spotted to capture the mRNA present in overlaid tissues. These positional barcodes enable the maintenance of positional information throughout the process of cDNA synthesis, library preparation, and sequencing. With decreasing spot diameters and spot distance, resolution will further increase, which is required for single cell analysis. A second approach to sequencing RNAs in the context of cells and tissues is fluorescent in situ sequencing (FISSEQ) (Lee et al., 2014). FISSEQ is a technology combining RNA‐FISH and next generation sequencing, allowing for detection of multiple RNAs at subcellular resolution. Where FISSEQ provides a higher resolution than spatial transcriptomics, it requires a panel of oligonucleotides to detect mRNAs of interest where spatial transcriptomics is unbiased and does not required a priori knowledge about genes of interest.

## Supporting information


**Figure S1** Distribution of reads mapping to different genomic regions, mitochondrial, and nuclear genes detected in microglia nuclear and cellular transcriptomes. **(a)** Distribution of confidentially mapped reads to exonic, intronic and intergenic regions for cells and nuclei in mouse (left panel) and human (right panel) samples. **(b)** Percentage of mitochondrial genes detected in cellular and nuclear data in mouse (left panel) and human (right panel) samples. **(c)** Percentage of ribosomal genes detected in cellular and nuclear data in mouse (left panel) and human (right panel) samples.

**Figure S2** Genes and counts per mouse cell/nucleus and expression of cell type specific markers. **(a)** PCA plot of all profiled cells and nuclei, the colors indicate different experimental samples. **(b)** UMAP depicting the number of UMI counts per cell/nucleus. **(c)** UMAP depicting the number of unique genes expressed per cell/nucleus. **(d)** UMAP depicting log expression values of *Hexb* (microglia), *Aqp4* (astrocytes), *Map1b* (neurons) and *Plp1* (oligodendrocytes), respectively.

**Figure S3** Genes and counts per human cell/nucleus for donors 1 and 2 combined. **(a)** UMAP depicting the number of UMI counts per cell/nucleus. **(b)** UMAP depicting the number of unique genes expressed per cell/nucleus. **(c)** UMAPs depicting log expression values of *CSF1R* (microglia), *AQP4* (astrocytes), *MAP1B* (neurons) and *PLP1* (oligodendrocytes), respectively.

**Table S1** Differential gene expression analysis between LPS and PBS treatment group in cells and nuclei from mouse bulk sequencing

**Table S2** GO analysis of the LPS responsive genes in cells and nuclei from mouse bulk sequencing

**Table S3** Differentially expressed gene analysis between cells and nuclei in PBS and LPS condition from mouse bulk sequencing

**Table S4** Differentially expressed gene analysis between PBS and LPS in cells and nuclei from mouse single cell/nucleus sequencing

**Table S5** Differentially expressed gene analysis between cells and nuclei in PBS and LPS condition from mouse single cell/nucleus sequencing

**Table S6** Differential expression analyisis between cells and fresh nuclei within each donor in single cell/nucleus squencing

## Data Availability

The data reported in this study are available through Gene Expression Omnibus at https://www.ncbi.nlm.nih.gov/geo with accession number GSE135618.
