# Supplementary figures and images for: Transcriptional profiling of microglia; current state of the art and future perspectives
Source: Glia. 2019 Dec 17;68(4):740–55. doi: 10.1002/glia.23767 (PMC7064956; doi:10.1002/glia.23767)

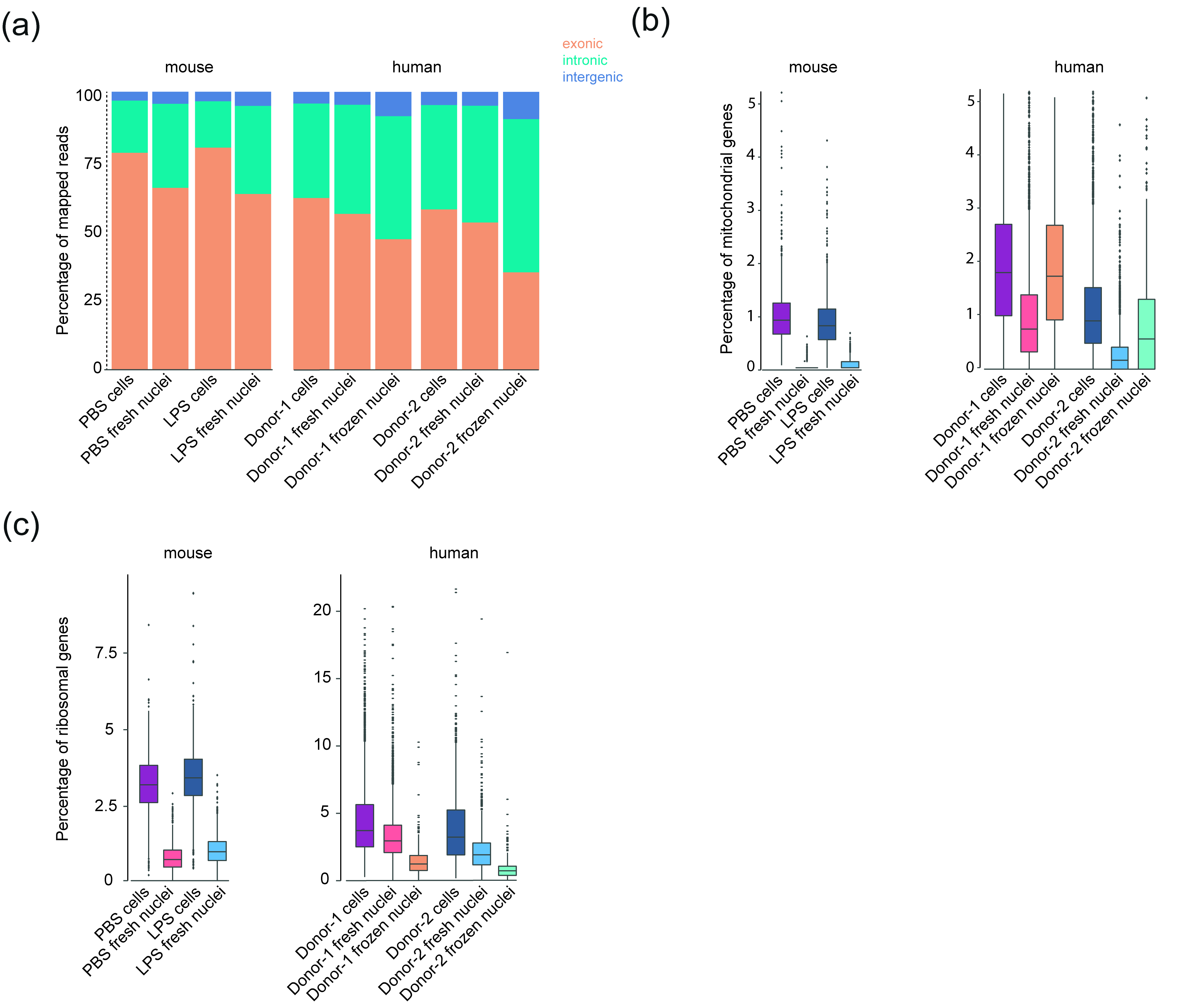

Supplement: Supplementary file 1 — Figure S1 Distribution of reads mapping to different genomic regions, mitochondrial, and nuclear genes detected in microglia nuclear and cellular transcriptomes. (a) Distribution of confidentially mapped reads to exonic, intronic and intergenic regions for cells and nuclei in mouse (left panel) and human (right panel) samples. (b) Percentage of mitochondrial genes detected in cellular and nuclear data in mouse (left panel) and human (right panel) samples. (c) Percentage of ribosomal genes detected in cellular and nuclear data in mouse (left panel) and human (right panel) samples. [file GLIA-68-740-s001.tif]

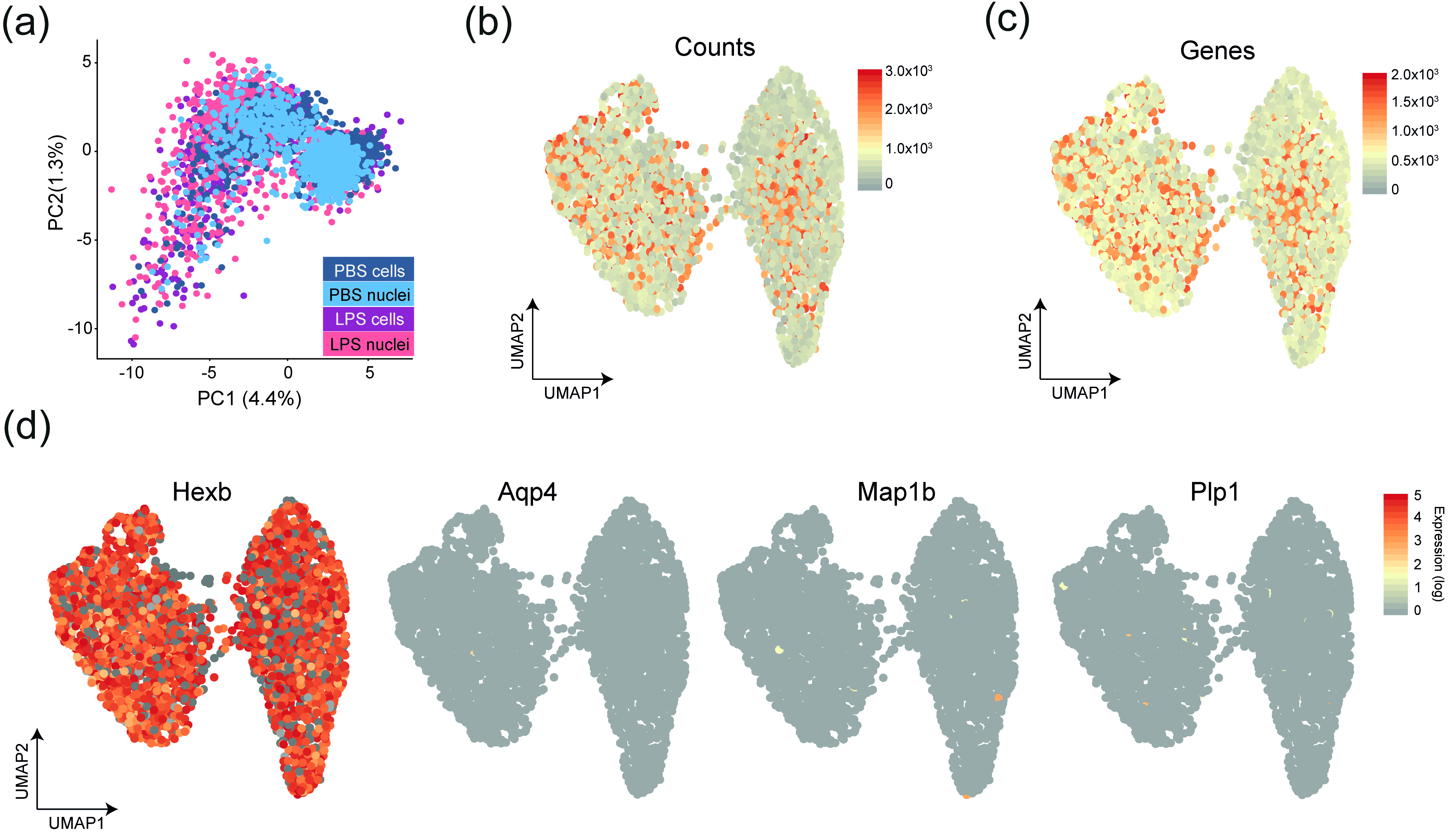

Supplement: Supplementary file 2 — Figure S2 Genes and counts per mouse cell/nucleus and expression of cell type specific markers. (a) PCA plot of all profiled cells and nuclei, the colors indicate different experimental samples. (b) UMAP depicting the number of UMI counts per cell/nucleus. (c) UMAP depicting the number of unique genes expressed per cell/nucleus. (d) UMAP depicting log expression values of Hexb (microglia), Aqp4 (astrocytes), Map1b (neurons) and Plp1 (oligodendrocytes), respectively. [file GLIA-68-740-s002.tif]

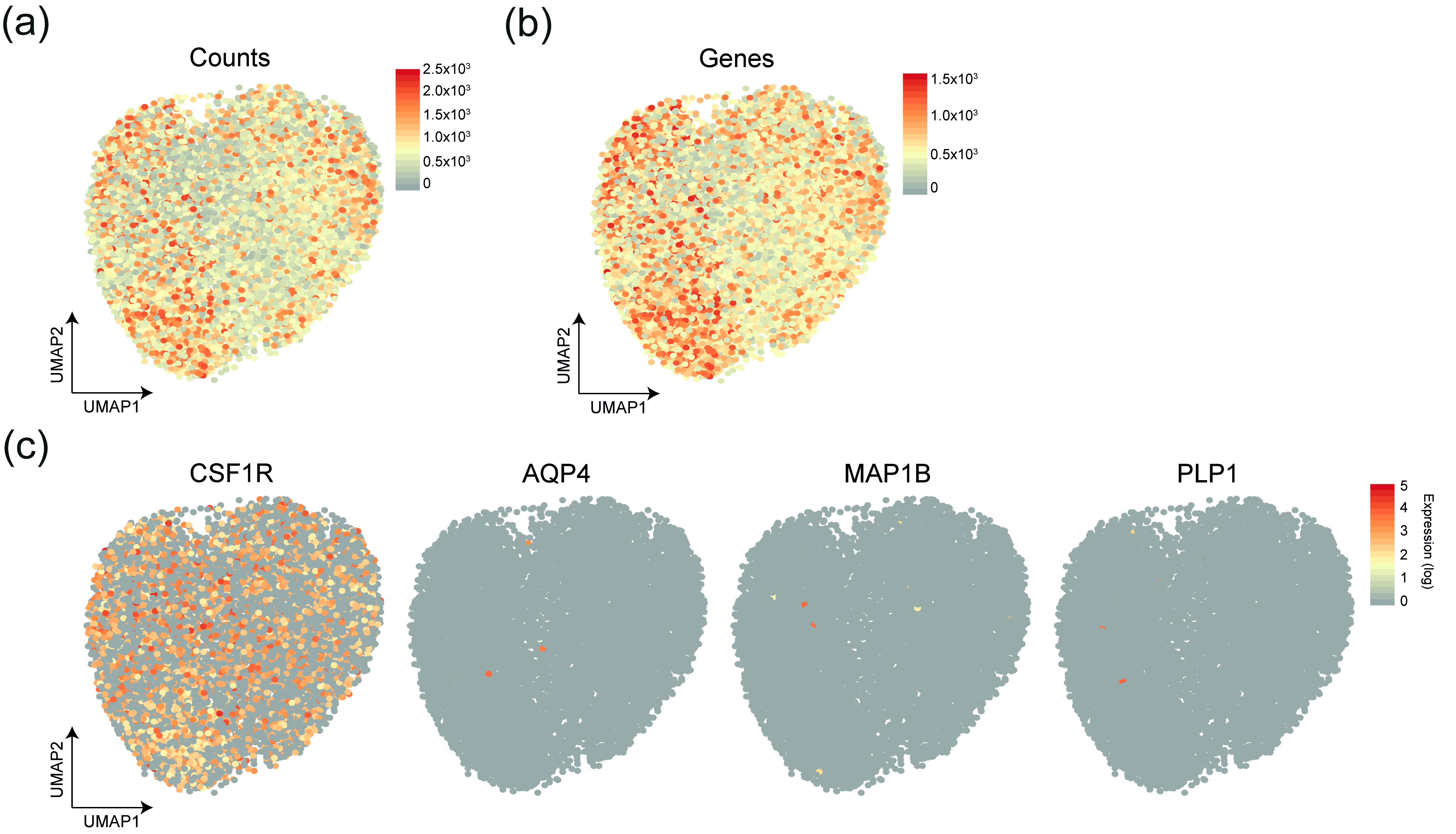

Supplement: Supplementary file 3 — Figure S3 Genes and counts per human cell/nucleus for donors 1 and 2 combined. (a) UMAP depicting the number of UMI counts per cell/nucleus. (b) UMAP depicting the number of unique genes expressed per cell/nucleus. (c) UMAPs depicting log expression values of CSF1R (microglia), AQP4 (astrocytes), MAP1B (neurons) and PLP1 (oligodendrocytes), respectively. [file GLIA-68-740-s003.tif]
